# Health Professions’ Digital Education: Review of Learning Theories in Randomized Controlled Trials by the Digital Health Education Collaboration

**DOI:** 10.2196/12912

**Published:** 2019-03-12

**Authors:** Shweta Bajpai, Monika Semwal, Ram Bajpai, Josip Car, Andy Hau Yan Ho

**Affiliations:** 1 Centre for Population Health Sciences Lee Kong Chian School of Medicine Nanyang Technological University Singapore Singapore; 2 Ageing Research Institute for Society and Education Nanyang Technological University Singapore Singapore; 3 Global eHealth Unit, Department of Primary Care and Public Health School of Public Health Imperial College London London United Kingdom; 4 Psychology Programme School of Social Sciences Nanyang Technological University Singapore Singapore; 5 Palliative Care Centre for Excellence in Research and Education Singapore Singapore

**Keywords:** learning theory, health professions, digital education, digital health education, digital education interventions

## Abstract

**Background:**

Learning theory is an essential component for designing an effective educational curriculum. Reviews of existing literature consistently lack sufficient evidence to support the effectiveness of digital interventions for health professions’ education, which may reflect disconnections among learning theories, curriculum design, use of technology, and outcome evaluation.

**Objective:**

The aim of this review was to identify, map, and evaluate the use of learning theories in designing and implementing intervention trials of health professions’ digital education, as well as highlight areas for future research on technology-enhanced education via the establishment of a development framework for practice and research.

**Methods:**

We performed a systematic search of Medical Literature Analysis and Retrieval System Online, Excerpta Medica database, Cochrane Central Register of Controlled Trials (Cochrane Library), PsycINFO, Cumulative Index to Nursing and Allied Health Literature, Education Resources Information Center, and Web of Science for randomized controlled trials (RCTs) published between 2007 and 2016.

**Results:**

A total of 874 RCTs on digital health education were identified and categorized into online-offline, mobile digital education, and simulation-based modalities for pre and postregistration health professions’ education. Of these, 242 studies were randomly selected for methodological review and thematic analysis. Data were extracted by one author using a standardized form, with a (48/242, 20%) random sample extracted by a second author, in duplicate. One-third (81/242, 33.4%) of the studies reported single or multiple learning theories in design, assessment, conceptualization, or interpretation of outcomes of the digital education interventions. Commonly reported learning theories were problem-based learning (16/81, 20%), social learning theory (11/81, 14%), and cognitive theory of multimedia learning (10/81, 12%). Most of these studies assessed knowledge (118/242, 48.8%), skills (62/242, 25.6%), and performance (59/242, 24.3%) as primary outcomes with nonvalidated assessment tools (151/242, 62.4%). Studies with reported learning theories (χ^2^_1_=8.2; *P*=.002) and validated instruments (χ^2^_1_=12.6; *P*=.006) have shown effective acquisition of learning outcomes.

**Conclusions:**

We proposed a Theory-Technology Alignment Framework to safeguard the robustness and integrity of the design and implementation of future digital education programs for the training of health professionals.

## Introduction

### Background

Digital education is one of the most popular and rapidly evolving approaches to teaching and learning in health professions’ education. It offers a wide range of intervention modalities using information and communication technologies, such as computer-assisted learning, mobile learning, and digital simulation–based learning, which enable individuals to acquire knowledge and skills in a timely and cost-effective manner with greater personal control [1,2]. The Mayo Clinic, a sprawling national health care system in the United States, reported that a sizeable portion of the national expenditure (approximately US $1 billion over 3 to 5 years) goes to digital medical education [3], and other advanced nations are following a similar developmental trend. Despite such huge investments, there is a lack of sufficient evidence to support the effectiveness of digital interventions for health professions’ education [4]. Well-conducted randomized controlled trials (RCTs) are the optimal design for evaluating the effectiveness of an intervention; this also applies to digital education interventions in health professions’ education. However, in the absence of clear theoretical foundations to measure change in learning outcomes, the methodological integrity of RCTs may be compromised, weakening the process of research, and ultimately weakening the validity of results [5-9].

Implementation of learning theories in the design of digital education could reduce such uncertainties as they provide structured theoretical and practical foundations that help educators develop the curricula, pedagogies, and assessments that are most relevant and conducive to student learning [10,11]. Learning theories also help learners understand their own learning processes, recognize ways to ensure short- and long-term maintenance of learning, and engage in effective practices to achieve intended learning outcomes [12-14]. Furthermore, learning theories inform and inspire pedagogy strategies that serve to ensure good teaching practices for both traditional and digital education, enabling educators to identify and understand the complexity and specificity of knowledge acquisition, in addition to providing insights for effective curriculum design and appropriate measurement of learning outcomes [15]. Although there is a wealth of learning theories available to help guide and evaluate traditional education designs, these theories have not been consistently applied or realized in the development of digital education [16]. Without a robust learning theory foundation or pedagogy framework to guide or evaluate digital health education, its effectiveness for achieving optimal learning outcomes is highly questionable [17,18]. In fact, an increasing body of evidence reveals that theory-based learning intervention has greater impact over nontheory-based interventions [12,19-24].

### Objectives

There is a paucity of research on the extent to which digital intervention for health professions’ education design integrates educational theory. The lack of understanding of how learners acquire knowledge via different digital modalities makes it difficult to determine the appropriate outcomes to measure when evaluating the effectiveness of such interventions. Therefore, this methodological review aimed to address this important but often neglected area of digital health professions’ education. We carried out a critical analysis of digital interventions within health professions’ education to determine the extent to which learning theories were explicitly used in the intervention design and evaluation and examine how these theories have been implemented. Finally, on the basis of the resulting findings, we conceptualized a development framework for augmenting learning theories in the design of digital interventions for health professions.

## Methods

### Defining Intervention Modalities

Digital education includes a variety of technologies such as offline and online computer-based digital education, digital game–based learning (DGBL), massive open online courses, virtual reality (VR), virtual patient simulation (VPS), psychomotor skills trainers, and mobile digital education [25]. For this study, we classified digital education into 3 broad sections on the basis of the nature of the educational content and delivery. We grouped studies that used online modes with those that used offline modes, such as CD-ROM or universal serial bus sticks, as *online-offline* interventions. Studies that used mobile phones, tablets, personal digital assistants, and other handheld devices for delivering educational content were grouped as *mobile digital education* interventions. Finally, studies that utilized simulation in the learning intervention, such as VR, VPS, and DGBL, were categorized as * digital* *simulation–based* *education.*

### Study Design and Data Sources

Our study is a methodological review that adopts both a quantitative and a qualitative evaluative approach. This review is part of a global evidence synthesis initiative on digital health professions’ education [26]. A systematic literature search for digital health education RCTs and quasi-experimental studies was carried out using the following databases from January 1990 to August 2016: Medical Literature Analysis and Retrieval System Online (Ovid), Excerpta Medica database (Elsevier), Cochrane Central Register of Controlled Trials (Cochrane Library, Wiley), PsycINFO (Ovid), Educational Research Information Centre (Ovid), Cumulative Index to Nursing and Allied Health Literature (EBSCO), and Web of Science Core Collection (Thomson Reuters; Multimedia Appendix 1).

### Study Selection

A total of 874 intervention studies, published between January 2007 and August 2016, on different areas of digital education, were identified through our database search. We included only pre- and post-registration health professions as listed in the Health Field Education and Training (091) of the International Standard Classification of Education (United Nations Educational, Scientific and Cultural Organization Institute for Statistics, 2013) [27]. Learners of traditional, alternative, and complementary medicine were excluded. A convenience sample of 25.2% (220/874) was drawn randomly from these studies to understand the reporting pattern of learning theories in digital education interventions. We selected only one-fourth of these studies in the methodological review, as our objective was to understand and identify the general trend of reporting, and such a sampling approach was deemed adequate and feasible in previous methodological review studies on education interventions [28]. We also extracted and included the data of 22 randomly selected pilot studies in the review to understand and highlight the reporting style of learning theories. Therefore, a total of 242 (220+22 pilot studies) unique studies were used in this analysis. Microsoft Excel was used to generate random numbers.

### Data Extraction

Data were extracted for each included study. We developed the data extraction form through pilot testing and revised it further according to the feedback from coauthors. We extracted information including random number, mode of digital learning, first author, year of publication, title of the study, name of the journal, sample size, study population, setting, country where research was originally conducted, primary outcomes, measurement instrument, validation of measurement instrument, and the theory mentioned in the included studies. Data were extracted by the first author (SB) and verified by the second author (MS), and discrepancies were resolved through discussion, with adjudication by a senior author (AH) when necessary.

### Identification and Analysis of Theories

This study investigated the use of learning theory reported in the interventions of 3 modalities of digital health professions’ education. To be judged as having a theory used, 3 criteria had to be met. First, any study that explicitly named a learning theory used in the design of intervention or learning evaluation was considered as theory used. Second, any study that described the use of pedagogy and the theoretical framework relevant to learning theory in intervention design and learning evaluation was considered as theory used. Third, any study that did not explicitly mention a learning theory but had clearly employed a learning theory in intervention design and learning evaluations was considered as theory used. In instances where a study merely mentioned that its intervention or evaluation design was based on *pedagogical or learning principles* but did not name the theory or describe its relevant features, was not considered as having used a theory and was excluded from this review. Decisions on theory used were based on consensus of the working group in case of uncertainty (SB, MS).

### Statistical Analysis

Data were directly entered into Microsoft Excel and subsequently cleaned for invalid entries. The analysis was mostly descriptive; we summarized data as frequency and percentage for categorical items and median and interquartile range (IQR) for continuous items. We analyzed predefined study characteristics of all included digital learning interventions as previously described in data extraction section. We quantified associations using the Chi-square test and 2-sided *P*<.05 was considered as statistical significance. All analyses were performed using Stata software (version 14.0, StataCorp).

## Results

### General Characteristics of Included Digital Education Intervention Studies

**Figure 1:**
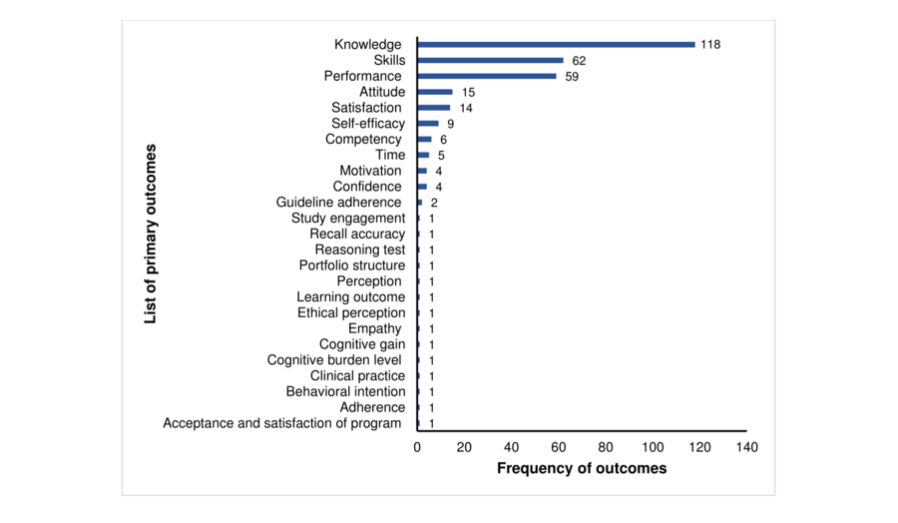
Frequency distribution of reported primary outcomes in digital health professions’ education intervention studies.

We evaluated 242 studies, published between 2007 and 2016, from 3 modalities of digital health professions’ education interventions: online-offline–based digital education (154/242, 63.6%), mobile digital education (21/242, 8.7%), and digital simulation–based education (67/242, 27.7%; see Table 1). Most of the studies were published between 2012 and 2016 (155/242, 64.0%) and conducted in high-income countries including the United States (102/242, 42.1%), United Kingdom (25/242, 10.3%), Germany (14/242, 5.7%), Canada (13/242, 5.3%), and Australia (11/242, 4.5%). The study population in the majority of the studies was preregistration health professionals (148/242, 61.1%) and the median (IQR) of the study size was 72 (43-120). Only one-third of the studies (81/242, 33.4%) mentioned any type of learning theory applied in the intervention design. More than half of the studies (151/242, 62.4%) used nonvalidated measurement instruments to assess primary outcomes. Most studies assessed knowledge (118/242, 48.7%), skills (62/242, 25.6%), and performance (59/242, 24.4%) as primary outcomes (Figure 1). The preferred choice of measurement structures was self-reported multiple-choice questions (MCQs; 69/242, 28.5%), questionnaire (66/242, 27.3%), scales (48/242, 19.8%), and a combination of the tools (18/242, 7.4%). However, in some studies (17/242, 7.0%) the measurement assessment tools were not clearly specified (Multimedia Appendix 2).

**Table 1:** Characteristics of included digital health professions’ education intervention studies.

Study characteristics			Type of digital domain			Total (N=242)
			Online-offline–based education (N=154)	Mobile digital education (N=21)	Digital simulation–based education (N=67)	
**Year of publication, n (%)**						
		2007	4 (2.6)	1 (4.7)	3 (4.4)	8 (3.3)
		2008	10 (6.5)	1 (4.7)	5 (7.4)	16 (6.6)
		2009	10 (6.5)	1 (4.7)	4 (5.9)	15 (6.2)
		2010	16 (10.4)	2 (9.5)	6 (8.9)	24 (9.9)
		2011	17 (11)	1 (4.7)	6 (8.9)	24 (9.9)
		2012	25 (16.2)	0 (0)	12 (17.9)	37 (15.2)
		2013	16 (10.3)	4 (19)	7 (10.4)	27 (11.1)
		2014	21 (13.6)	3 (14.2)	9 (13.4)	33 (13.6)
		2015	22 (14.2)	4 (19)	10 (14.9)	36 (14.8)
		2016	13 (8.4)	4 (19)	5 (7.4)	22 (9)
**Type of population, n (%)**						
		Undergraduate	86 (55.8)	14 (66.6)	5 (7.4)	148 (61.1)
		Postgraduate	45 (29.2)	5 (23.8)	14 (20.9)	64 (26.4)
		Mixed population	23 (14.9)	2 (9.5)	48 (71.6)	30 (12.4)
**Setting, n (%)**						
	Hospital		60 (38.9)	10 (47.6)	16 (23.8)	86 (35.5)
	University		94 (61)	11 (52.3)	51 (76.1)	156 (64.4)
Study size, median (interquartile range)			84 (47-138)	63 (42-72)	52 (30-93)	72 (43-120)
**Top 5 countries of publication, n (%)**						
	United States		69 (44.8)	8 (38)	25 (37.3)	102 (42.2)
	United Kingdom		20 (12.9)	0 (0)	5 (7.4)	25 (10.3)
	Germany		10 (6.5)	0 (0)	4 (5.9)	14 (5.7)
	Canada		10 (6.5)	1 (4.7)	2 (2.9)	13 (5.3)
	Australia		6 (3.9)	1 (4.7)	4 (5.9)	11 (4.5)
**Statistical significance of primary outcomes, n (%)**						
	No		74 (48)	8 (38)	25 (37.3)	107 (44.2)
	Yes		71 (46.1)	11 (52.3)	37 (55.2)	119 (49.1)
	Mixed		9 (5.8)	2 (9.5)	5 (7.4)	16 (6.6)
**Reported validity of the instrument used, n (%)**						
	No		86 (55.8)	16 (76.1)	49 (73.1)	151 (62.4)
	Yes		68 (44.1)	5 (23.8)	18 (26.8)	91 (37.6)
**Reported learning theory for the design of intervention, n (%)**						
	No		99 (64.2)	12 (57.1)	50 (74.6)	161 (66.5)
	Yes		55 (35.7)	9 (42.8)	17 (25.3)	81 (33.4)

### Categorization of Reported Learning Theories in Digital Education Interventions

In 33.5% (81/242) studies that reported learning theories, a total of 42 theories were applied in the design of digital health professions’ education interventions (Table 2). The most commonly applied learning theories were problem-based learning (16/81, 20%), social learning theory (11/81, 14%), Mayer's cognitive theory of multimedia learning (10/81, 12%), and adult learning theory (8/81, 10%). Furthermore, 7 theories including cognitive load theory (7/81, 9%), Kirkpatrick's framework (6/81, 7%), cognitive theory of learning (6/81, 7%), constructive theory of learning (5/81, 6%), Bloom’s taxonomy (5/81, 6%), collaborative learning (5/81, 6%), and social cognitive learning (4/81, 5%) were employed 5 to 7 times across different intervention studies. The remaining 32 theories were sparsely reported. In terms of theory application and integration, 48 out of 81 studies (59%) reported only 1 theory in the design of the digital education programs, 22 out of 81 studies (27%) reported integrating 2 theories, and 8 out of 81 studies (10%) used 3 to 6 theories to develop the interventions.

Given the vast number of theories reported, we further organized each theory into a set of thematic categories on the basis of its nature and characteristics. Among the 42 identified theories, only 13 referred to a specific learning theory. Of these, 7 theories were categorized under cognitivism, which focuses on the inner mental activity and information processing of learners. Furthermore, 3 theories were organized into the constructivism category. The remaining 3 theories were standalone idiosyncratic models of learning. A total of 9 theories were categorized under design-based learning, which comprises a combination of multiple learning theories for explaining learning processes and pedagogy practices. A total of 9 theories were categorized under behavior-change theories that explain the processes of health-related behavioral and attitudinal transformation. Furthermore, 3 theories were organized into the social sciences category, 3 into the decision-making and therapeutic framework category, 3 into the learning style category, and 2 theories into the motivational theory category. (Multimedia Appendix 3).

**Table 2:** List and frequency of reported learning theories (n=42) by modality in digital health professions’ education intervention studies (total reported studies=81).

Name of theory	Online-offline–based education (n=55), n (%)	Mobile digital education (n=9), n (%)	Digital simulation–based education (n=17), n (%)	Total (N=81), n (%)
Problem-based learning	11 (20)	1 (11.1)	5 (29.4)	17 (21)
Social learning theory	9 (16.3)	0 (0)	2 (11.7)	11 (13.5)
Mayer’s cognitive theory of multimedia learning	4 (7.2)	3 (33.3)	3 (17.6)	10 (12.3)
Adult learning theory	7 (12.7)	1 (11.1)	0 (0)	8 (9.8)
Cognitive load	2 (3.6)	1 (11.1)	4 (23.5)	7 (8.6)
Kirkpatrick's framework	6 (10.9)	0 (0)	0 (0)	6 (7.4)
Cognitive theory	3 (5.4)	2 (22.2)	1 (5.8)	6 (7.4)
Constructive theory	4 (7.2)	1 (11.1)	0 (0)	5 (6.1)
Bloom’s taxonomy	3 (5.4)	1 (11.1)	1 (5.8)	5 (6.1)
Collaborative learning	5 (9)	0 (0)	0 (0)	5 (6.1)
Social cognitive theory	4 (7.2)	0 (0)	0 (0)	4 (4.9)
Theory of self-efficacy	4 (7.2)	0 (0)	0 (0)	4 (4.9)
Information processing	1 (1.8)	1 (11.1)	1 (5.8)	3 (3.7)
Health belief model	3 (5.4)	0 (0)	0 (0)	3 (3.7)
Situated learning	2 (3.6)	0 (0)	1 (5.8)	3 (3.7)
Dual coding theory	1 (1.8)	2 (22.2)	0 (0)	3 (3.7)
Kolb's experiential learning	0 (0)	0 (0)	3 (17.6)	3 (3.7)
Innovation diffusion theory	2 (3.6)	0 (0)	0 (0)	2 (2.4)
Cooperative learning	1 (1.8)	0 (0)	1 (5.8)	2 (2.4)
Social constructivism	0 (0)	1 (11.1)	1 (5.8)	2 (2.4)
Theory of reasoned action	2 (3.6)	0 (0)	0 (0)	2 (2.4)
Cognitive dissonance theory	2 (3.6)	0 (0)	0 (0)	2 (2.4)
Cognitive apprenticeship model	1 (1.8)	0 (0)	0 (0)	1 (1.2)
Theory of behavior change	1 (1.8)	0 (0)	0 (0)	1 (1.2)
Cognitive flexibility theory	1 (1.8)	0 (0)	0 (0)	1 (1.2)
Cognitive behavioral therapy theory	1 (1.8)	0 (0)	0 (0)	1 (1.2)
Enquiry-based learning	1 (1.8)	0 (0)	0 (0)	1 (1.2)
Practice-based learning	1 (1.8)	0 (0)	0 (0)	1 (1.2)
Theory of reflective practice	1 (1.8)	0 (0)	0 (0)	1 (1.2)
Bowen's teaching strategy	1 (1.8)	0 (0)	0 (0)	1 (1.2)
Banning's theoretical framework	0 (0)	1 (11.1)	0 (0)	1 (1.2)
Positive psychological theoretical framework	0 (0)	0 (0)	1 (5.8)	1 (1.2)
System approach model	1 (1.8)	0 (0)	0 (0)	1 (1.2)
Persuasive communication model	1 (1.8)	0 (0)	0 (0)	1 (1.2)
Social support theory	1 (1.8)	0 (0)	0 (0)	1 (1.2)
Social marketing theory	1 (1.8)	0 (0)	0 (0)	1 (1.2)
Theory of self-determination	1 (1.8)	0 (0)	0 (0)	1 (1.2)
Wittrock’s generative learning theory	1 (1.8)	0 (0)	0 (0)	1 (1.2)
Elaboration theory	1 (1.8)	0 (0)	0 (0)	1 (1.2)
Taxonomy of significant learning	0 (0)	0 (0)	1 (5.8)	1 (1.2)
ARCS model of motivational design^a^	1 (1.8)	0 (0)	0 (0)	1 (1.2)
Connectivism	1 (1.8)	0 (0)	0 (0)	1 (1.2)

^a^This model is based on four steps for promoting and sustaining motivation in the learning process: attention, relevance, confidence, and satisfaction.

### Comparison of Learning Theory Used With Study Characteristics and Thematic Analysis

There were no significant differences in the publication years, types of digital domain, research settings, sample population, and sample size among studies that did or did not report learning theory used. However, studies that reported the validated measurement instrument (χ^2^_1_=12.6; *P*=.006) and significant primary outcomes (χ^2^_1_=8.2; *P*=.002) were statistically associated with the reporting of learning theory (Multimedia Appendix 4).

Furthermore, thematic analysis revealed that out of the 81 studies that reported learning theories, 70% (57/81) of the studies were judged to have a relatively clear use of theory in interventions (Multimedia Appendix 5). Among these 57 studies, 27 studies clearly provided the description of use of theory to develop an instructional design, 10 studies used theory-based assessment tools to measure learning outcomes, 4 studies used a theoretical framework to evaluate learning system, and 4 studies used theory to justify their findings. Another 4 studies used theory only for developing study hypothesis and objectives, but the effects were not observed. Furthermore, 7 other studies simply stated that a theory was used in the conceptualization of the intervention without a clear description, whereas 1 study reported partial use of theory to develop the intervention. On the other hand, 7 out of 81 studies (9%) reported learning theory but did not provide any explicit explanation about theory usability in methods and only mentioned a theory name in the introduction and discussion sections. Moreover, 17 out of 81 studies (21%) only named a learning theory vaguely or implicitly; therefore, inference was drawn on the basis of the available description on the theory used. Finally, from conceptualization to conclusion of results, no study was completely based on a learning theory framework.

A closer examination of different digital modalities revealed that among offline-online intervention studies, (n=55), 37 out of 55 studies (67%) clearly elaborated on the purpose of the theory used, 29 out of 55 studies (53%) used validated measurement instruments to assess learning outcomes, and 17 out of 55 studies (31%) reported effectiveness of digital interventions compared with the conventional method of learning. Among the mobile digital intervention studies, all included studies (n=9) discussed the purpose of the theory used, 3 studies used a validated instrument to assess the outcomes, and 2 studies reported significant results. Among the digital simulation–based intervention studies (n=17), 11 studies out of 17 (65%) were judged to have employed theory in their interventions, 9 studies assessed outcomes implying valid tools, and 5 studies reported statistically significant results (Multimedia Appendix 5).

## Discussion

### Principal Findings

This is the first ever study that comprehensively reviewed the application of learning theory within the design and evaluation of digital health professions’ education interventions, inclusive of online-offline–based education, mobile digital education, and digital simulation–based education modalities. Our analyses highlight 4 serious concerns that may hamper the developmental integrity of this fast growing industry, including (1) design and implementation of digital health professions’ education interventions without integration of appropriate pedagogy frameworks (161/242, 66.5%), (2) poor selection of learning theories for developing or supporting education interventions, (3) not selecting appropriate theory(s) for different modalities of digital health education such as computer-assisted learning, Web-based learning, mobile learning, and others, and (4) inappropriate usage of learning theory(s) and a nonvalidated assessment instrument that results in the mismatch of learning outcomes. The issues identified with the lack of learning theories and inappropriate application of learning theories to the development of digital education for health professions impede high-quality research into the efficacy of digital learning in this area.

Specifically, our findings reveal that the interventions for health professions’ education did not utilize a learning theory or pedagogical framework in curriculum design, program implementation, or learning evaluation. In fact, only one-third of the intervention studies included in our review were informed by learning theories; nevertheless, the purpose of theory utilization was unclear in many instances. This phenomenon may be explained by the intentionality of interventions. All published articles focused primarily on the use of technology and its effectiveness on learning rather than carefully applying learning theory and pedagogy in the design of digital curriculums [18]. Consequently, most studies are comparative in nature, with an emphasis to contrast technology-assisted education with traditional education methods in producing learning outcomes, ignoring the appropriate use of learning theories for education design [29,30] and failing to adequately investigate the mechanisms that make digital education effective.

We also observed a lack of clarity and explanations for the selection of learning theories and pedagogy that informed digital education design, implementation, and evaluation [24]. Poor selection of theory could be a result of nonavailability of reporting guidelines [16]. Given the vast number of learning theories available in existing literature with overlapping characteristics, it may prove difficult to choose a specific theory for developing an effective curriculum while keeping a specific set of learners in mind [31,32]. It is also important to note that no learning theory has been established precisely for illuminating the inner working of digital education, despite rapid developments in the field, and this could result in overt simplification of the use of new technology to shape digital education without truly realizing its impact on learning and pedagogy [18,20,33-35]. Unmistakably, a comprehensive understanding of technological prowess combined with relevant and well-versed learning principles is urgently needed to improve the quality of education delivered to digital learners [36,37].

Another important finding derived from our review is the improper selection and reporting of measurement tools and the use of a nonvalidated instrument. Although certain psychological or attitudinal constructs of learning outcomes, such as self-efficacy, often demand the use of specific self-assessment instruments, validation may not always be feasible or practical. In addition, nonvalidated self-assessment tools have also been widely criticized for their lack of accuracy [38]. It is thus recommended that if validation has not been carried out for an established scale at the time of education intervention, especially that related to psychological and attitudinal constructs, then confirmatory factor analysis needs to be conducted upon data collection to ensure validity and reliability of instrument used [39].

Moreover, most studies in our review assessed knowledge followed by skills and performance as primary outcomes. We observed a high degree of incongruities (71/81, 88%) among the choice of learning outcomes, the underlying learning theory, and the use of measurement instruments for assessing such learning outcomes (measurement instruments; Multimedia Appendix 5). In most of the studies, the measurement tools do not adequately fit the learning outcomes of the reported learning theory. For instance, it is difficult, if not impossible, to assume that MCQs in nonstandardized tests (151/242, 62.4%), which are strongly associated with assessing lower cognitive processing such as fact recall [40], can adequately assess problem-solving skills, knowledge application, motivation, teamwork, and creativity, all of which are primary learning outcomes of the most frequently reported learning theories in our review. Specifically, problem-based learning, the top reported learning theory in our review, aims to foster teamwork and creative real-world problem-solving abilities [41]; on the other hand, social learning theory, the second most reported theory, aims to produce behavioral, motivational, and attitudinal changes [42]; finally, Mayer’s cognitive theory of multimedia learning, the third most reported theory, aims to enhance information processing and creative thinking [43]. As learning outcomes are used and interpreted in various ways, it is important to choose an appropriate, valid, and theory-based instrument that essentially assesses what it intended to assess [44]. Poor evaluation processes in digital health professions’ education can compromise curriculum design, mislead learning outcomes [24], and lead to poor clinical practices, which ultimately put patient care at stake.

Notably, our review showed significant association among the application of learning theory, validity of the instrument used, and statistical significance of primary outcomes. Findings revealed that studies that did not apply learning theory (110/161, 68.3%) were significantly less likely to use validated measurement instrument. Similarly, primary outcomes were statistically significant in a majority of the studies (52/81, 64%) that applied learning theory and validated assessment tools. In addition, more than half of the studies reporting a nonvalidated assessment tool may indicate a poor understanding of validity theory (ie, what constitutes a valid outcome measure) and poor contextualization and application of validity and reliability in medical education [45]. Therefore, the design of the intervention from hypothesis to measuring outcomes could be improved when the research begins with an appropriate theory or pedagogical framework.

### Limitations of the Study

Despite the important findings reported, this review comprises some limitations. First, this is not a systematic review of theory-used in digital learning, which might increase the likelihood of missing some important study(s) pertaining to the topic. Second, we reviewed only one-fourth of published studies from 3 modalities; however, digital learning includes various modalities, but feasibility restriction does not permit us to include all published studies from different modalities of digital education. Nonetheless, future research may aim to focus on these issues, including proper reporting of theory-used and measurement tools for assessment. Given the multimodal nature of digital education, it is recommended that unless we have the solid theoretical guidelines for development, implementation, and evaluation, we are unlikely to achieve the desired learning goals [16].

**Figure 2:**
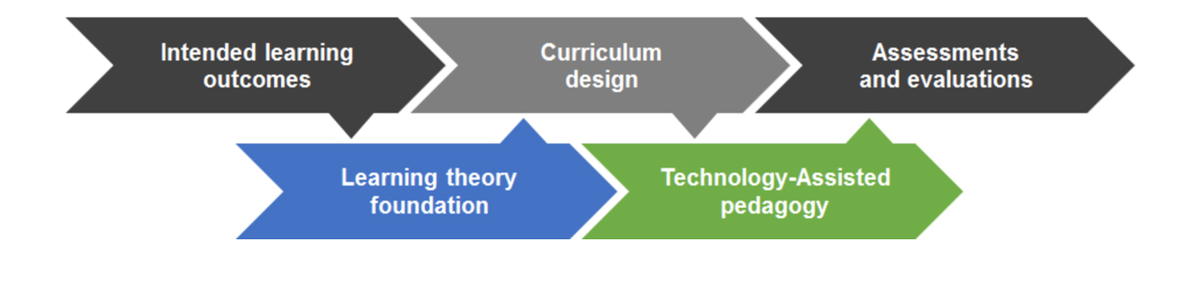
Theory-Technology Alignment Framework for health professions’ digital education.

### Theory-Technology Alignment Framework

The appropriate application of learning theories in digital education is essential to ensure curriculum integrity and critical to successful learning outcomes [46,47]. Therefore, we outlined a Theory-Technology Alignment Framework (TTAF) to inform the development of digital education for health professionals (Figure 2). In conventional practices of designing teaching, learning, and assessment activities [48], educators begin with a set of *intended learning outcomes* that they would like students to acquire upon the completion of education and training. On the basis of these outcomes, they proceed to *curriculum design* by incorporating various teaching and learning activities that they believe would lead to the intended learning outcomes, and thereafter the *assessments and evaluations* procedures are established to evaluate whether students have attained the intended outcomes, as well as assess standards of performance. We argue that this widely adopted practice known as constructive alignment [49] is too simplistic in the context of digital education design and neglects the intricate connections and interplays among learning processes, technology, and pedagogy practices. We propose that to achieve a holistic learning experience via digital education, intended learning outcomes and curriculum design must be informed and aligned by an appropriate *learning theory foundation*, one that includes a collection of the most relevant learning theories to ensure the effective choice and application of teaching and learning activities. This would empower educators to better conceptualize and create a conducive pedagogical framework and effective learning environment to help students achieve success. Once a fitting teaching framework and learning environment are clearly delineated and established, they can then be augmented by using appropriate *technology-assisted pedagogy*, which effectively aligns with and supports learning theory foundation and curriculum design for developing theory-driven assessments and evaluations for adequately measuring students’ performance in accordance to the original intended learning outcomes.

Putting the TTAF in practice, a digital education intervention for medical trainees may aspire toward the *intended learning outcomes* of (1) evidence-based clinical diagnosis with (2) team-based problem solving. To achieve these outcomes, one can apply a *learning theory foundation* that incorporates team-based learning theory with the Mayer’s cognitive theory of multimedia learning to inform *curriculum design*. This may comprise pedagogical instructions that utilize visual and auditory information processing for making proper patient diagnosis in a team-based environment. Furthermore, a set of theory-informed teaching and learning activities, matching *technology-assisted pedagogy*, such as the use of digital patient records including medical charts, x-rays, and computed tomography scans, coupled with voice recordings of patient intake assessments and patient-physician communications, can be used to facilitate the first intended learning outcome. Moreover, an online discussion forum with a built-in monitoring and feedback mechanism for course instructors can be created for trainee groups to deliberate on the digital patient medical information they have and to share their analysis of problem, to come to a joint diagnosis; this will serve to facilitate the second intended learning outcome. With this alignment and integration of curriculum design and technology-assisted pedagogy, an appropriate *assessments and evaluations* protocol can be developed to ensure that all intended learning outcomes are properly assessed. To assess the first outcome, evaluation on individual trainee’s understanding and application of digital patient medical information in clinical diagnosis may be conducted through short-answer questions and higher order MCQs that test the cognitive processes of information analysis and knowledge application. To assess the second outcome, continuous monitoring of team-based discussion, as well as evaluation of written reflections on group processes, and group-based problem-solving capacity via stimulated online clinical meetings may be conducted.

In short, we recommend that instead of placing technology at the center of digital education, one must begin at the fundamental roots of learning theory and pedagogy. Therefore, the TTAF could be useful for designing an effective digital education intervention.

### Conclusions

Our study has opened the doors for future research by highlighting many problems in digital health professions’ education research and its different modalities. This multifaceted problem can be tackled through effective utilization of appropriate learning theories to foster stronger integration among intended outcomes, curriculum design, pedagogy activities, and evaluations with pertinent computer-assisted technologies. Imperatively, high-quality digital research using a clear theoretical framework, well-defined outcomes, and standardized assessment tools with adequate validity evidences is urgently warranted with proper reporting in methodology. Our review serves as an important guideline for researchers, educators, policy makers, and program designers to develop an effective intervention for the training of health professionals, and if applied proficiently, it would assist in advancing the field of digital research in medical education by addressing the various methodological shortcomings that exist in current interventional studies.

## Appendix

Multimedia Appendix 1 MEDLINE (Ovid) search strategy.

Multimedia Appendix 2 Frequency distribution of reported measurement instruments in digital medical education intervention studies.

Multimedia Appendix 3 Categorization and theoretical description of reported learning theories and related theories in digital medical education intervention studies (n=42).

Multimedia Appendix 4 Comparison of study characteristics with the reporting of learning theory in digital medical education intervention studies.

Multimedia Appendix 5 Thematic analysis of theory used in digital health professions’ education intervention studies (n=81).

## Abbreviations

DGBL: digital game–based learning
IQR: interquartile range
MCQs: multiple-choice questions
RCT: randomized controlled trials
TTAF: Theory-Technology Alignment Framework
VPS: virtual patient simulation
VR: virtual reality
