# Supplementary material for: Health Professions’ Digital Education: Review of Learning Theories in Randomized Controlled Trials by the Digital Health Education Collaboration
Source: J Med Internet Res. 2019 Mar 12;21(3):e12912. doi: 10.2196/12912 (PMC6434396; doi:10.2196/12912)
Supplement: Multimedia Appendix 1 [file jmir_v21i3e12912_app1.docx]

**Appendix 1: MEDLINE (Ovid) search strategy**

1. exp education, professional/ not education, veterinary/

2. Education, Predental/

3. Education, Premedical/

4. exp Students, Health Occupations/

5. ((medic* or premedic* or dent* or laborator* or predent* or midwi?e* or nurs* or nutrition* or orthop* or podiat* or pharmac* or psycholog* or psychiatr* or health or healthcare or occupational therap* or physiotherap* or physical therap* or clinical or surg* or radiolog* or obstetric* or gyn?ecolog* or orthodont* or An?esthesi* or Dermatolog* or Oncolog* or Rheumatolog* or Neurolog* or Patholog* or P?ediatric* or Cardiolog* or Urolog*) adj3 (student* or graduate* or undergraduate* or staff or personnel or practitioner* or clerk* or fellow* or internship* or residen* or educat* or train* or novice* or tutor*)).tw,kf.

6. 1 or 2 or 3 or 4 or 5

7. Computer-Assisted Instruction/

8. exp Internet/

9. Computer Simulation/

10. Patient Simulation/

11. software/

12. Mobile Applications/

13. User-Computer Interface/

14. Video Games/

15. Web Browser/

16. Education, Distance/

17. Computers/

18. exp Microcomputers/

19. exp Cell Phones/

20. Games, Experimental/

21. exp Models, Anatomic/

22. Audiovisual Aids/

23. Educational Technology/

24. Electronic Mail/

25. exp Telemedicine/

26. Telenursing/

27. Telecommunications/

28. Webcasts/

29. exp Videoconferencing/

30. ((computer* or digital* or hybrid or blended or mixed mode or distance or remote* or electronic or mobile or online* or interactiv* or multimedia or internet or web* or virtual* or game* or gaming or Videogame* or Videogaming) adj3 (classroom* or course* or educat* or instruct* or learn* or lecture* or simulat* or train* or teach* or tutor* or platform*)).tw,kf.

31. (Simulat* adj3 (course* or educat* or instruct* or learn* or train* or teach* or platform* or high-fidelity)).tw,kf.

32. e-learn*.tw,kf.

33. elearn*.tw,kf.

34. m-learn*.tw,kf.

35. mlearn*.tw,kf.

36. smartphone*.tw,kf.

37. smart-phone*.tw,kf.

38. ((mobile or cell) adj2 phone*).tw,kf.

39. iphone*.tw,kf.

40. android*.tw,kf.

41. ipad*.tw,kf.

42. Personal digital assistant*.tw,kf.

43. handheld computer*.tw,kf.

44. Mobile App?.tw,kf.

45. Mobile Application?.tw,kf.

46. webcast*.tw,kf.

47. webinar*.tw,kf.

48. flipped classroom*.tw,kf.

49. Serious game*.tw,kf.

50. Serious gaming.tw,kf.

51. Patient Simulat*.tw,kf.

52. Virtual patient*.tw,kf.

53. ((educat* or instruct* or learn* or simulat* or train* or teach* or interactiv*) adj2 technolog*).tw,kf.

54. Massive Open Online Course?.tw,kf.

55. Mooc?.tw,kf.

56. (Canvas network or Coursera or Coursesites or edx or Futurelearn or iversity or miriada x or moodle or novoed or openlearning or open2study or plato or spoc or udacity or pingpong).tw,kf.

57. 7 or 8 or 9 or 10 or 11 or 12 or 13 or 14 or 15 or 16 or 17 or 18 or 19 or 20 or 21 or 22 or 23 or 24 or 25 or 26 or 27 or 28 or 29 or 30 or 31 or 32 or 33 or 34 or 35 or 36 or 37 or 38 or 39 or 40 or 41 or 42 or 43 or 44 or 45 or 46 or 47 or 48 or 49 or 50 or 51 or 52 or 53 or 54 or 55 or 56

58. 6 and 57

59. Education.fs.

60. Education/

61. Teaching/

62. Learning/

63. exp Inservice Training/

64. Curriculum/

65. educat*.tw,kf.

66. learn*.tw,kf.

67. train*.tw,kf.

68. instruct*.tw,kf.

69. teach*.tw,kf.

70. 59 or 60 or 61 or 62 or 63 or 64 or 65 or 66 or 67 or 68 or 69

71. Health Personnel/

72. exp Allied Health Personnel/

73. Anatomists/

74. "Coroners and Medical Examiners"/

75. exp Dental Staff/

76. exp Dentists/

77. Health Educators/

78. Infection Control Practitioners/

79. Medical Laboratory Personnel/

80. exp Medical Staff/

81. exp Nurses/

82. exp Nursing Staff/

83. Personnel, Hospital/

84. Pharmacists/

85. exp Physicians/

86. Physician*.tw,kf.

87. Doctor*.tw,kf.

88. Nurs*.tw,kf.

89. Surg*.tw,kf.

90. Health Personnel.tw,kf.

91. healthcare professional*.tw,kf.

92. radiolog*.tw,kf.

93. dentist*.tw,kf.

94. Pharmacist*.tw,kf.

95. Hospital Administrator*.tw,kf.

96. Podiatr*.tw,kf.

97. Psycholog*.tw,kf.

98. Psychiatr*.tw,kf.

99. An?esthesi*.tw,kf.

100. Clinician*.tw,kf.

101. Dermatolog*.tw,kf.

102. General practioner*.tw,kf.

103. Cardiolog*.tw,kf.

104. Oncolog*.tw,kf.

105. Rheumatolog*.tw,kf.

106. Neurolog*.tw,kf.

107. Patholog*.tw,kf.

108. P?ediatric*.tw,kf.

109. Physiotherap*.tw,kf.

110. Physical therap*.tw,kf.

111. Occupational therap*.tw,kf.

112. dieti?ian*.tw,kf.

113. Dietetic*.tw,kf.

114. midwi?e*.tw,kf.

115. nutrition*.tw,kf.

116. orthopti*.tw,kf.

117. obstetric*.tw,kf.

118. gyn?ecolog*.tw,kf.

119. orthodont*.tw,kf.

120. Urolog*.tw,kf.

121. 71 or 72 or 73 or 74 or 75 or 76 or 77 or 78 or 79 or 80 or 81 or 82 or 83 or 84 or 85 or 86 or 87 or 88 or 89 or 90 or 91 or 92 or 93 or 94 or 95 or 96 or 97 or 98 or 99 or 100 or 101 or 102 or 103 or 104 or 105 or 106 or 107 or 108 or 109 or 110 or 111 or 112 or 113 or 114 or 115 or 116 or 117 or 118 or 119 or 120

122. Health Occupations/

123. exp Allied Health Occupations/

124. Biomedical Engineering/

125. Chiropractic/

126. exp Dentistry/

127. exp Evidence-Based Practice/

128. exp Medicine/

129. exp Nursing/

130. Dietetics/

131. Optometry/

132. Orthoptics/

133. exp Pharmacology/

134. exp Pharmacy/

135. Podiatry/

136. Psychology, Medical/

137. Serology/

138. Specialization/

139. exp Surgical Procedures, Operative/

140. exp Radiography/

141. 122 or 123 or 124 or 125 or 126 or 127 or 128 or 129 or 130 or 131 or 132 or 133 or 134 or 135 or 136 or 137 or 138 or 139 or 140

142. 121 or 141

143. 57 and 70 and 142

144. Psychomotor Performance/

145. motor skills/

146. ((psychomotor or procedural or technical) adj3 skill*).tw,kf.

147. (psychomotor adj3 performance).tw,kf.

148. 144 or 145 or 146 or 147

149. 6 and 148

150. 58 or 143 or 149

151. randomized controlled trial.pt.

152. controlled clinical trial.pt.

153. randomized.ab.

154. placebo.ab.

155. drug therapy.fs.

156. randomly.ab.

157. trial.ab.

158. groups.ab.

159. 151 or 152 or 153 or 154 or 155 or 156 or 157 or 158

160. exp animals/ not humans.sh.

161. 159 not 160

162. 150 and 161

163. limit 162 to yr="1990 -Current"
