# Supplementary material for: Health Professions’ Digital Education: Review of Learning Theories in Randomized Controlled Trials by the Digital Health Education Collaboration
Source: J Med Internet Res. 2019 Mar 12;21(3):e12912. doi: 10.2196/12912 (PMC6434396; doi:10.2196/12912)

**Appendix 2: Frequency distribution of reported measurement instruments in digital medical education intervention studies**


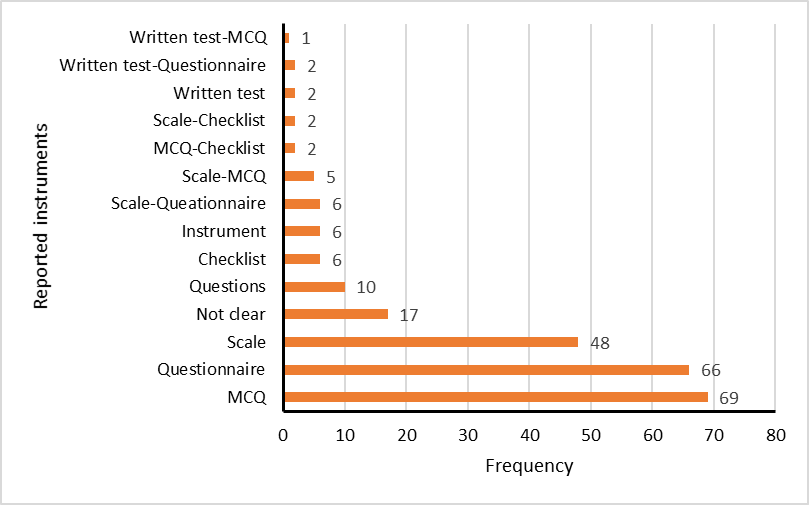

Supplement: Multimedia Appendix 2 [file jmir_v21i3e12912_app2.docx]
