# Supplementary material for: Health Professions’ Digital Education: Review of Learning Theories in Randomized Controlled Trials by the Digital Health Education Collaboration
Source: J Med Internet Res. 2019 Mar 12;21(3):e12912. doi: 10.2196/12912 (PMC6434396; doi:10.2196/12912)
Supplement: Multimedia Appendix 3 [file jmir_v21i3e12912_app3.docx]

**Appendix 3: Categorisation and theoretical description of reported learning theories and related theories in digital medical education intervention studies (n=42)**

| **Paradigm** | **Name of theory** | **Name of theorist (year)** | **Description of the theories** |
| --- | --- | --- | --- |
| **Learning theory group** |  |  |  |
| **Cognitivism** | Cognitive theory | Piaget 1936; Tolman, 1948; Miller 1956; Atkinson and Shiffrin 1968 | Focuses on the inner mental activities, way of processing information, mode of thinking, remembering and reorganizing and problem solving. |
|  | Information processing theory | Miller 1956; Atkinson and Shriffin, 1968 | Information processing theory discusses the mechanisms through which learning occurs. Specifically, it focuses on aspects of memory encoding and retrieval. |
|  | Dual coding theory | Paivio, 1986 | A theory of cognition according to which humans process and represent verbal and non-verbal information in separate, related systems. |
|  | Cognitive load | Sweller, 1988 | Theory employs aspects of information processing theory to emphasize the inherent limitations of concurrent working memory load on learning during instruction |
|  | Cognitive apprenticeship model | Collins,1989 | A theory that attempts to bring tacit processes out in the open.  It assumes that people learn from one another, through observation, imitation and modelling |
|  | Cognitive flexibility theory | Spiro and Jehng, 1992 | The ability to spontaneously restructure one's knowledge, in many ways, in adaptive response to radically changing situational demands. |
|  | Mayer’s cognitive theory of multimedia learning | Mayer, 2009 | Based on three main assumptions: there are two separate channels (auditory and visual) for processing information; there is limited channel capacity; and that learning is an active process of filtering, selecting, organizing, and integrating information. |
| **Constructivism** | Constructive theory | Dewey 1916; Piaget 1972; Vygotsky 1978; and Bruner 1973 | People actively construct or create their own subjective representations of objective reality, based on a learner’s prior experience. |
|  | Theory of reflective practice | Schon, 1983 | Learning to reflect in action (RIA) and look back on action (ROA) together form a reflective process for decision-making and professional growth. |
|  | Social constructivism | McKinley, 2015 | A sociological theory of knowledge according to which human development is socially situated and knowledge is constructed through interaction with others. |
| **Adult learning** | Adult learning theory | Knowles, 1984 | Based on 4 assumptions (andragogy) - self-concept, Adult learner experience, readiness to learn, orientation to learning, motivation to learn. |
| **Situated learning** | Situated learning theory | Lave and Wenger, 1991 | Focus the relationship between learning and the social situation in which it occurs. |
| **Connectivism learning** | Connectivism  theory | Siemens, 2005 | Connectivism is the integration of principles explored by chaos, network, and complexity and self-organization theories. |
| **Design-based learning** | Kirkpatrick’s model | Kirkpatrick, 1950 | It considers the value of any type of training, formal or informal, across four levels. Reaction, learning, behaviour and results. |
|  | Bloom's Taxonomy | Bloom, 1956 | A set of three hierarchical models used to classify educational learning objectives into levels of complexity and specificity. |
|  | Wittrock’s generative Learning Theory | Wittrock, 1974 | Based on the idea that learners can actively integrate new ideas into their memory to enhance their educational experience. |
|  | Elaboration theory | Reigeluth,1979 | An instructional design theory that argues that content to be learned should be organized from simple to complex order, while providing a meaningful context in which subsequent ideas can be integrated. |
|  | Experiential learning | Kolb, 1984 | The process of learning through experience, and is more specifically defined as "learning through reflection on doing’. |
|  | System approach model | Dick and Carey, 1990 | The model addresses instruction as an entire system, focusing on the interrelationship between context, content, learning and instruction. |
|  | Problem based learning | Savery, 1995 | A student-cantered pedagogy in which students learn about a subject through the experience of solving an open-ended problem found in trigger material. |
|  | Enquiry based learning | University of Manchester, 2012 | Includes [problem-based learning](https://en.wikipedia.org/wiki/Problem-based_learning), and is generally used in small scale investigations and projects, as well as [research](https://en.wikipedia.org/wiki/Research). |
|  | Taxonomy of significant learning | Fink, 2013 | The expression of learning outcomes in terms of his six-part taxonomy. Foundational Knowledge understanding and remembering information and ideas, human dimension - learning about oneself, others. |
| **Behaviour-change theories** | Theory of behavior change |  | Attempts to explain why behaviours change. These theories cite environmental, personal, and behavioural characteristics as the major factors in behavioural determination. |
|  | Health belief model | Hochbaum,1950s; Rosenstock, 1974 | A psychological model that attempts to explain and predict health behaviours. This is done by focusing on the attitudes and beliefs of individuals. |
|  | Cognitive dissonance theory | Festinger, 1957 | States that a powerful motive to maintain cognitive consistency can give rise to irrational and sometimes maladaptive behaviour. |
|  | Cognitive behavioral therapy theory | Beck, 1960s | A short-term, goal-oriented psychotherapy treatment that takes a hands-on, practical approach to problem-solving. (Rakovshik, 2013). |
|  | Theory of reasoned action | Fishbein and Ajzen, 1975 | Predicts that behavioural intent is created or caused by two factors: attitudes and subjective norms. Attitudes have two components and called as evaluation and strength of a belief. |
|  | Theory of self-efficacy | Bandura, 1977 | Self-efficacy as one's belief in one's ability to succeed in specific situations or accomplish a task. |
|  | Persuasive communication model | Shrigley, 1978 | An approach for attitude change. |
|  | Social learning theory | Bandura, 1977 | Theory posits that people learn from one another, via observation, imitation, and modelling. |
|  | Social cognitive theory | Bandura, 1986 | The theory states that when people observe a model performing a behaviour and the consequences of that behaviour, they remember the sequence of events and use this information to guide subsequent behaviours |
| **Social Sciences Theory** | Innovation diffusion theory | Rogers, 1962 | It originated in communication to explain how, over time, an idea or product gains momentum and diffuses (or spreads) through a specific population or social system. |
|  | Social support theory | Glanz, 2002 | Social networks are linkages between people that may provide social support and that may serve functions other than providing support. |
|  | Social marketing theory | International Social Marketing Association, 2013 | Social marketing is the use of marketing theory, skills and practices to achieve social change. |
| **Decision-making and therapeutic framework** | Positive psychology theoretical framework | Seligman, 2003 | Positive psychology provides a different perspective on motivation, emotion, and ultimately decision making, and in so doing contributes to a balanced understanding of consumer behaviour. |
|  | Banning’s theoretical framework | Banning, 2003 | A theoretical framework of applied pharmacology and therapeutics. |
|  | Bowen’s teaching strategy | Bowen, 2006 | Assess a learner’s diagnostic reasoning strategies. |
| **Learning style** | Collaborative learning | Dillenbourg, 1999 | A situation in which two or more people learn or attempt to learn something together. |
|  | Cooperative learning | Johnson 2009 | An educational approach which aims to organize classroom activities into academic and social learning experiences. |
|  | Practice-based learning | Moore, 2003 | Practice-based learning and improvement (PBLI) describes a collection of activities that physicians and other members of the health care team engage in to link opportunities for improvement with resources that can address those opportunities. |
| **Motivational theories** | ARCS model of motivational design | Keller, 1979 | Based on four steps for promoting and sustaining motivation in the learning process: Attention, relevance, confidence, and satisfaction. |
|  | Theory of self-determination | Ryan, 2002 and Deci, 2012 | It is concerned with supporting our natural or intrinsic tendencies to behave in effective and healthy ways. |
