# Supplementary material for: Health Professions’ Digital Education: Review of Learning Theories in Randomized Controlled Trials by the Digital Health Education Collaboration
Source: J Med Internet Res. 2019 Mar 12;21(3):e12912. doi: 10.2196/12912 (PMC6434396; doi:10.2196/12912)
Supplement: Multimedia Appendix 4 [file jmir_v21i3e12912_app4.docx]

**Appendix 4: Comparison of study characteristics with the reporting of learning theory in digital medical education intervention studies**

| **Characteristics** | **Learning theory** | | **Combined**  **(n = 242)** | **p-value** |
| --- | --- | --- | --- | --- |
|  | **Reported**  **(n = 81)** | **Not-reported**  **(n = 161)** |  |  |
| **Year of publication** |  |  |  | 0.987 |
| 2007 - 2010 | 21 (25.9) | 42 (26.0) | 63 (26.0) |  |
| 2011 - 2013 | 29 (35.9) | 59 (36.7) | 88 (36.4) |  |
| 2014 - 2016 | 31 (38.3) | 60 (37.3) | 91 (37.6) |  |
| **Type of digital domain** |  |  |  | 0.207 |
| eLearning | 55 (67.9) | 99 (61.5) | 154 (63.6) |  |
| mLearning | 9 (11.1) | 12 (7.5) | 21 (8.7) |  |
| Simulation-based learning | 17 (21.0) | 50 (31.0) | 67 (27.7) |  |
| **Type of journal** |  |  |  | 0.976 |
| General | 24 (29.6) | 48 (29.8) | 72 (29.8) |  |
| Specific | 57 (70.4) | 113 (70.2) | 170 (70.3) |  |
| **Type of population** |  |  |  | 0.425 |
| Graduate | 46 (56.8) | 102 (63.4) | 148 (61.2) |  |
| Postgraduate | 22 (27.1) | 42 (26.0) | 64 (26.4) |  |
| Mixed population | 13 (16.1) | 17 (10.6) | 30 (12.4) |  |
| **Setting** |  |  |  | 0.360 |
| Hospital | 32 (39.5) | 54 (33.5) | 86 (35.5) |  |
| University | 49 (60.5) | 107 (66.5) | 156 (64.5) |  |
| **Study size** |  |  |  | 0.515 |
| <100 | 53 (65.4) | 112 (69.6) | 165 (68.2) |  |
| ≥100 | 28 (34.6) | 49 (30.4) | 77 (31.8) |  |
| **Reported validity of instrument**  **used** | |  |  | 0.007 |
| No | 41 (50.6) | 110 (68.3) | 151 (62.4) |  |
| Yes | 40 (49.4) | 51 (31.7) | 91 (37.6) |  |
| **Statistical significance of results** | |  |  | 0.002 |
| No | 23 (28.4) | 84 (52.2) | 107 (44.2) |  |
| Yes | 52 (64.2) | 67 (41.6) | 119 (49.2) |  |
| Mixed | 6 (7.4) | 10 (6.2) | 16 (6.6) |  |
