# Supplementary material for: Health Professions’ Digital Education: Review of Learning Theories in Randomized Controlled Trials by the Digital Health Education Collaboration
Source: J Med Internet Res. 2019 Mar 12;21(3):e12912. doi: 10.2196/12912 (PMC6434396; doi:10.2196/12912)
Supplement: Multimedia Appendix 5 [file jmir_v21i3e12912_app5.docx]

### Appendix 5: Thematic analysis of theory used in digital health professions’ education intervention studies (n = 81)

| **Modality** | **Author, year** | **Name of theory** | **Purpose of theory used** | **Outcome** | **Measurement instrument** | **Validity** | **Result** |
| --- | --- | --- | --- | --- | --- | --- | --- |
| Mobile digital education | Lee,  2016 | ARCS model of motivational design | To measure the outcome | Motivation, competency, satisfaction | Questionnaire | Yes | Significant |
| Mobile digital education | Kruck, 2016 | Mayer’s cognitive theory of multimedia learning, cognitive load | Conceptualized by theory | Knowledge | MCQ | Yes | Significant |
| Mobile digital education | Lee, 2015 | Cognitive and constructive learning theory, problem-based | Conceptualized by theory | Motivation, learning readiness | Scale | No | Significant |
| Mobile digital education | Gadbury-Amyot, 2014 | Dual code, cognitive theory of multimedia learning | Objective on the basis of theory | Skills | Scale | No | Not significant |
| Mobile digital education | De Oliveira, 2013 | Adult learning theory | To support results | Performance | Scale | No | Significant |
| Mobile digital education | Pimmer, 2013 | Dual code, Meyer’s cognitive theory of multimedia learning | Hypothesis on the basis of theory | Knowledge, recall | Scale | No | Mixed results |
| Mobile digital education | Chaung, 2013 | Banning’s theoretical framework, information processing | Framework on the basis of theory | Knowledge | MCQ | No | Significant |
| Mobile digital education | Hansen, 2011 | Mayer's cognitive theory of multimedia learning | Framework on the basis of theory | Skills, confidence | Questionnaire | No | Significant |
| Mobile digital education | Johnston, 2010 | Bloom's taxonomy, social constructive theory | Framework on the basis of theory | Performance | Questionnaire | Yes | Not significant |
| Online-offline–based | de Beurs, 2016 | Adult learning, diffusion of innovation | Model on the basis of theory | Confidence, knowledge, guideline adherence | Questionnaire | Yes | Not significant |
| Online-offline–based | Richmond, 2016 | Problem-based | Not clear | Knowledge, skills, self-efficacy, attitude, satisfaction | MCQ | No | Mixed results |
| Online-offline–based | Tai, 2015 | Collaborative learning | Conceptualized by theory | Performance | MCQ | No | Significant |
| Online-offline–based | Moreira, 2015 | Kirkpatrick framework | Evaluation on the basis of framework | Knowledge, satisfaction | Questionnaire | No | Significant |
| Online-offline–based | Dolan, 2015 | Cognitive dissonance | To support results | Knowledge | MCQ | Yes | Significant |
| Online-offline–based | de Beurs, 2015 | Adult learning theory, diffusion of innovation | Model on the basis of theory | Guideline adherence | Questionnaire | Yes | Significant |
| Online-offline–based | Gartmeier, 2015 | Social learning (role model), problem-based | Not clear | Knowledge, competency | Scale | Yes | Significant |
| Online-offline–based | Esche, 2015 | Kirkpatrick framework | Framework on the basis of theory | Knowledge, satisfaction | Scale | No | Significant |
| Online-offline–based | Marshall, 2014 | Social learning (role play) | Not clear | Satisfaction | Scale | No | Significant |
| Online-offline–based | Yang, 2014 | Mayer’s cognitive theory of multimedia learning | Not clear | Performance | MCQ | No | Significant |
| Online-offline–based | Hsu, 2014 | Bloom’s taxonomy | To measure the outcome | Communication competency, knowledge, satisfaction, self-efficacy | Scale, checklist | Yes | Mixed results |
| Online-offline–based | Fisher, 2014 | Adult learning theory | Not clear | Perception, skills | Questionnaire | Yes | Significant |
| Online-offline–based | Kontio, 2014 | Reflective learning | Intervention on the basis of theory | Knowledge | Questionnaire | No | Not significant |
| Online-offline–based | Chang, 2014 | Cognitive load, Mayer’s cognitive theory of multimedia learning | Instructional design on the basis of theories | Knowledge | MCQ | No | Significant |
| Online-offline–based | Tung, 2014 | Health belief model, persuasive communication model, social cognitive theory, social support theory, social marketing theory, theory of self-efficacy | Framework on the basis of theories | Knowledge (awareness toward infectious and biological hazards) | Questionnaire | Yes | Significant |
| Online-offline–based | Harned, 2014 | Kirkpatrick framework | Theories mentioned in discussion section only | Knowledge, satisfaction, attitude, self-efficacy | Scale | Yes | Significant |
| Online-offline–based | Hayes, 2014 | Social cognitive learning, situated learning, adult learning | Conceptualized by theory | Skills | Questionnaire | Yes | Significant |
| Online-offline–based | Akar, 2014 | Health belief model | To measure the outcome | Knowledge, performance | Questionnaire | Yes | Significant |
| Online-offline–based | Bernstein, 2013 | Kirkpatrick framework | Evaluation on the basis of framework | Knowledge, clinical performance, confidence | MCQ | No | Significant |
| Online-offline–based | Wang, 2013 | Social learning (role model) | Not clear | Knowledge | Scale | Yes | Not significant |
| Online-offline–based | Rakovshik, 2013 | Kirkpatrick framework, problem-based, social learning (role play), cognitive behavioral therapy theory | Evaluation on the basis of framework | Skills, knowledge | Questionnaire | No | Significant |
| Online-offline–based | Lin, 2013 | Cognitive learning, cooperative learning | To measure the outcome | Skills, performance, knowledge | Questionnaire | Yes | Not significant |
| Online-offline–based | Mager, 2013 | Cognitive learning, theory of self-efficacy | To measure the outcome | Knowledge, confidence, self-efficacy | MCQ | Yes | Significant |
| Online-offline–based | Alfieri, 2012 | Kirkpatrick Framework | Evaluation on the basis of framework | Knowledge, skills | Questionnaire | Yes | Significant |
| Online-offline–based | Beidas, 2012 | Social learning (role play), problem-based, enquiry-based learning, collaborative learning | Not clear | Knowledge, skills, adherence, satisfaction | MCQ, checklist | Yes | Not significant |
| Online-offline–based | Chanakit, 2012 | Bloom’s taxonomy | Program on the basis of theory | Knowledge, skills, satisfaction | MCQ | Yes | Not significant |
| Online-offline–based | Ally, 2012 | Bloom’s taxonomy, elaboration theory | Objective on the basis of taxonomy | Knowledge | MCQ | No | Significant |
| Online-offline–based | Subramanian, 2012 | Cognitive apprenticeship model | Teaching strategies on the basis of model | Knowledge | MCQ | No | Significant |
| Online-offline–based | Kalet, 2012 | Wittrock’s theory of generative learning, Mayer’s cognitive theory of multimedia learning, constructive theory, dual code, cognitive load | Theories mentioned in introduction section only | Knowledge, skills | MCQ, checklist | Yes | Not significant |
| Online-offline–based | Irvine, 2012 | Social cognitive, theory of reasoned action, social learning, theory of self-efficacy | To measure the outcome | Knowledge, behavioral intention, attitude, self-efficacy | Scale | Yes | Significant |
| Online-offline–based | Bennett-Lev, 2012 | Problem-based | Not clear | Knowledge, skills, confidence, utilization | Questionnaire | Yes | Significant |
| Online-offline–based | Maloney, 2012 | Constructive theory | Teaching strategy on the basis of theory | Knowledge, satisfaction, | MCQ | No | Significant |
| Online-offline–based | Irvine, 2012 | Social cognitive, theory of reasoned action | To measure the outcome | Attitude, knowledge, self-efficacy | Scale | Yes | Mixed results |
| Online-offline–based | Dimeff, 2011 | Mayer’s cognitive theory of multimedia learning, situated learning | Not clear | Knowledge, self-efficacy | Questionnaire | Yes | Not significant |
| Online-offline–based | Carney, 2011 | Adult learning theory | Intervention on the basis of theory | Knowledge, satisfaction, performance (recall rates) | Questionnaire | No | Not significant |
| Online-offline–based | Mahnken, 2011 | Theory of self-determination | Hypothesis on the basis of theory | Knowledge | MCQ | No | Significant |
| Online-offline–based | Clement, 2011 | Social contact theory, cognitive dissonance | To support results | Knowledge, attitude | Scale | Yes | Significant |
| Online-offline–based | Marsh-Tootle, 2011 | Cognitive flexibility theory, behavior change theory, system approach, reflective practice, social cognitive theory, practice based | Program on the basis of theories | Knowledge | Questionnaire | No | Significant |
| Online-offline–based | Smeekens, 2011 | Theory of self-efficacy | To measure the outcome | Performance, self-efficacy | Checklist | Yes | Significant |
| Online-offline–based | Cox, 2011 | Adult learning theory | Framework on the basis of theory | Knowledge | MCQ | Yes | Significant |
| Online-offline–based | Lee, 2010 | Bowen's teaching strategy, problem-based | Teaching strategies on the basis of framework | Skills, performance, satisfaction | Questionnaire | Yes | Not significant |
| Online-offline–based | El Saadawi, 2010 | Problem-based | Instructional design on the basis of theories | Performance | Not clear | No | Significant |
| Online-offline–based | Raupach, 2010 | Problem-based | Theory mentioned in introduction section only | Knowledge, performance | Scale | No | Significant |
| Online-offline–based | Le, 2010 | Adult learning, Mayer’s cognitive theory of multimedia learning | Included some elements of theory | Knowledge, attitude, satisfaction | Scale | No | Significant |
| Online-offline–based | Locatis, 2010 | Collaborative learning | Conceptualized by theory | Learning outcomes | MCQ | No | Not significant |
| Online-offline–based | Schreiber, 2010 | Mayer’s cognitive theory of multimedia learning, connectivism | Theories mentioned in introduction section only | Knowledge | MCQ | No | Not significant |
| Online-offline–based | McGregor, 2010 | Collaborative learning, constructive theory | Not clear | Skills | Questionnaire | Yes | Not significant |
| Online-offline–based | Weingardt, 2009 | Social learning (role play) | Not clear | Knowledge, self-efficacy | Questionnaire | No | Not significant |
| Online-offline–based | Kutob, 2009 | Health belief model | Framework on the basis of theory | Performance | Questionnaire | Yes | Significant |
| Online-offline–based | Glicksman, 2009 | Adult learning theory | Not clear | Skills | Scale, checklist | Yes | Significant |
| Online-offline–based | Hull, 2009 | Constructive learning | Not clear | Skills | Questionnaire | No | Significant |
| Online-offline–based | Tunuguntla, 2008 | Cognitive learning | To measure the outcome | Competency, cognitive burden, time | MCQ | Yes | Not significant |
| Online-offline–based | Benjamin, 2008 | Social learning (role model) | Not clear | Knowledge | MCQ | No | Significant |
| Online-offline–based | Jenkins, 2008 | Problem-based | Teaching strategies on the basis of framework | Performance | MCQ | No | Not significant |
| Online-offline–based | Vash, 2007 | Problem-based | Program on the basis of problem-based learning | Knowledge, skills | MCQ, written test | No | Mixed results |
| Digital simulation–based | Rasmussen, 2016 | Cognitive load theory | Theory mentioned in introduction section only | Performance | Not clear | No | Significant |
| Digital simulation–based | Dankbaar, 2016 | Cognitive load | To measure the outcome | Skills, motivation | Questionnaire | Yes | Not significant |
| Digital simulation–based | Heist, 2016 | Cognitive load | To support results | Skills, time | Questionnaire | Yes | Mixed results |
| Digital simulation–based | Argenton, 2015 | Problem-based, positive psychology theoretical framework | Process on the basis of theory | Competency, performance | Questionnaire | Yes | Significant |
| Digital simulation–based | Bryant, 2015 | Situated learning | Conceptualized by theory | Performance, confidence | Questionnaire | Yes | Not significant |
| Digital simulation–based | Rogers, 2014 | Problem-based | Not clear | Knowledge, reasoning test | MCQ, scale | No | Significant |
| Digital simulation–based | Menzel, 2014 | Cooperative learning | Conceptualized by theory | Attitude | Scale | Yes | Significant |
| Digital simulation–based | Al-Dahir, 2014 | Social constructive theory, problem-based | Theory mentioned in introduction section only | Knowledge | MCQ | Yes | Significant |
| Digital simulation–based | Stefaniak, 2014 | Kolb's experiential learning, cognitive theory, cognitive load, information processing | Theory mentioned in introduction section only | Knowledge | MCQ | No | Significant |
| Digital simulation–based | Farra, 2013 | Situated learning | Framework on the basis of theory | Knowledge | MCQ | Yes | Significant |
| Digital simulation–based | Cook, 2012 | Problem-based | Process on the basis of theory | Performance | MCQ | No | Significant |
| Digital simulation–based | Robinson,2011 | Taxonomy of significant learning, Bloom’s taxonomy | Model on the basis of theory | Knowledge, skills | MCQ | No | Significant |
| Digital simulation–based | Estevez, 2010 | Social learning (modelling) | Not clear | Performance | Questionnaire | No | Significant |
| Digital simulation–based | Raupach, 2009 | Collaborative learning | Teaching model on the basis of theory | Skills | Scale | No | Not significant |
| Digital simulation–based | O’Connor, 2009 | Kolb's experiential learning | Not clear | Performance | MCQ | Yes | Significant |
| Digital simulation–based | Youngblood, 2008 | Kolb's experiential learning | Training method on the basis of theory | Performance | Scale | No | Significant |
| Digital simulation–based | Butow, 2008 | Social learning (role play), problem- based | Intervention on the basis of theories | Skills | Questionnaire | Yes | Not significant |
